# Identifying Key Themes of Care Coordination for Patients with Chronic Conditions in Singapore: A Scoping Review

**DOI:** 10.3390/healthcare11111546

**Published:** 2023-05-25

**Authors:** Chuan De Foo, Jia Yin Yan, Audrey Swee Ling Chan, Jason C H Yap

**Affiliations:** Saw Swee Hock School of Public Health, National University of Singapore and National University Health System, Singapore 117549, Singapore; jiayin_yan@u.nus.edu (J.Y.Y.); jasonyap@nus.edu.sg (J.C.H.Y.)

**Keywords:** care coordination, Singapore, scoping review

## Abstract

A projected rise in patients with complex health needs and a rapidly ageing population will place an increased burden on the healthcare system. Care coordination can bridge potential gaps during care transitions and across the care continuum to facilitate care integration and the delivery of personalised care. Despite having a national strategic vision of improving care integration across different levels of care and community partners, there is no consolidation of evidence specifically on the salient dimensions of care coordination in the Singapore healthcare context. Hence, this scoping review aims to uncover the key themes that facilitate care coordination for patients with chronic conditions in Singapore to be managed in the community while illuminating under-researched areas in care coordination requiring further exploration. The databases searched were PubMed, CINAHL, Scopus, Embase, and Cochrane Library. Results from Google Scholar were also included. Two independent reviewers screened articles in a two-stage screening process based on the Cochrane scoping review guidelines. Recommendation for inclusion was indicated on a three-point scale and rating conflicts were resolved through discussion. Of the 5792 articles identified, 28 were included in the final review. Key cross-cutting themes such as having standards and guidelines for care programmes, forging stronger partnerships across providers, an interoperable information system across care interfaces, strong programme leadership, financial and technical resource availabilities and patient and provider-specific factors emerged. This review also recommends leveraging these themes to align with Singapore’s national healthcare vision to contain rising healthcare costs.

## 1. Introduction

The burden brought about by an ageing population is a health system issue that countries are facing globally. The global population aged over 60 is expected to nearly double from 2015 to 2050, with a jump from 12% to 22% [1]. Health systems need to be prepared to manage the swell in chronic diseases attributed to this greying population. Singapore anticipates that one in four residents will be above the age of 65 by 2030, and an old age support ratio of 2.7 [2]. At present, 80% of the total disease burden is attributable to chronic conditions, with three in four aged 65 and above diagnosed with one of the three most common chronic conditions of diabetes, hypertension or high cholesterol, and more than 50% aged 60 and above have more than two such conditions [3,4,5]. 

As the needs of the population increase in complexity, there is a need to integrate health and social care for patients with multiple chronic conditions. Patients with more than three chronic conditions are 40% more likely to face issues with care coordination as compared to patients suffering from just one [6]. Furthermore, patients in the community face increasing difficulties accessing chronic disease-related care services other than their regular follow-up care [7]. 

The government’s aspirations of anchoring care in the community birthed the national strategy of the *Three Beyonds* promulgated in 2017, which aims to nurture a sustainable health ecosystem through the three prongs of *Beyond Healthcare to Health, Beyond Quality to Value and Beyond Hospital to Community* [8]. The overarching objectives of the *Three Beyonds* are to contain tertiary care utilisation, deliver cost-effective care and promote patient-centred comprehensive care. This was recently augmented by the Healthier SG strategic vision to be launched in stages from mid-2023, which aims to put primary care at the centre by strengthening links to other levels of care and community providers [9]. This shift firmly positions care coordination at the forefront of this upcoming health system transformation.

Although care coordination has been introduced in Singapore for more than ten years, no available study exists to review the elements of the implemented programmes. Beyond the Singapore context, several studies were conducted to identify the commonalities among programmes focusing on care coordination activities to determine key elements of care coordination that contribute towards improved patient care [10,11]. By identifying the components of effective care coordination, there is huge potential to improve the quality of care, enhance the patient experience and reduce costs to the health system, among other benefits, when patients are managed adequately as they transition across care interfaces [12,13,14,15]. 

Despite the centrality of care coordination, it has no globally agreed-upon definition. It is generally accepted that care coordination involves two or more parties who arrange patient care activities to facilitate the appropriate delivery of care [16]. Specific to this scoping review, care coordination is operationally defined as activities that bridge potential gaps in chronic disease care transitions to facilitate care integration and delivery of appropriate care targeted at patients’ needs [17,18,19,20]. Aligned with Singapore’s national healthcare strategies, this review will focus on care coordination programmes for community-dwelling patients suffering from chronic diseases.

Broadly, Singapore’s health system is dominated by medically oriented care coordination models with more recent shifts towards patient-centred holistic care. An example is reflected by the comprehensive and coordinated care seen in patient-centred medical homes (PCMHs) offering a suite of services for patients with selected chronic conditions [21]. To that end, the underlying value of care coordination is to offer medical, social and emotional support for care recipients, which can, if implemented in a patient-centric manner, allay patient anxiety, reduce unnecessary healthcare utilisation, and improve clinical outcomes [19,22].

In Singapore, the Agency of Integrated Care (AIC) oversees the majority of care integration in the public healthcare system and manages the funding provided by the Ministry of Health (MOH) for care coordination programmes. Despite having a national strategic vision of improving care integration across different levels of care and community partners, there is no consolidation of evidence specifically on the salient dimensions of care coordination in the Singapore context. Hence, this scoping review aims to identify the key themes that facilitate care coordination activities for patients with chronic conditions in Singapore to be managed in the community while illuminating under-researched areas in care coordination requiring further exploration. 

## 2. Materials and Methods

**Figure 1:**
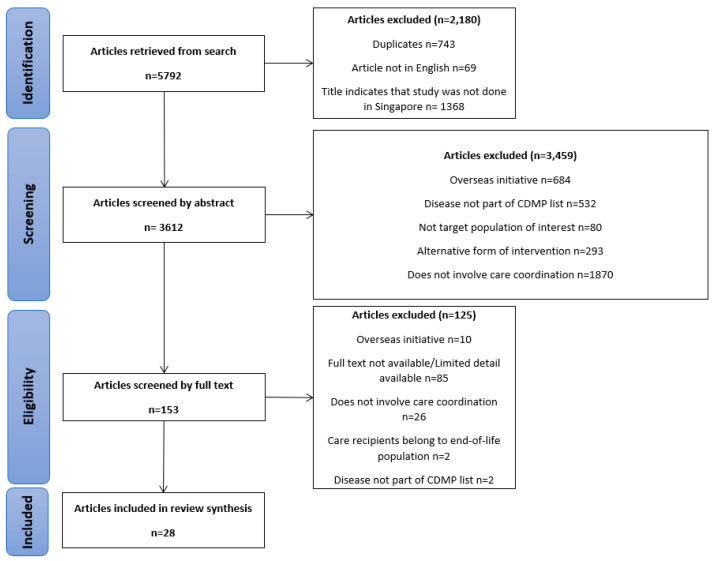
PRISMA flow chart illustrating search strategy used to identify eligible studies for inclusion.

This scoping review was conducted in accordance with the Preferred Reporting Items for Systematic Reviews and Meta-Analyses (PRISMA) and with a flow diagram (Figure 1). The PRISMA-ScR in Appendix A guided the entire scoping review process [23].

### 2.1. Search Strategy 

A systematic search of five databases (PubMed, CINAHL, Scopus, Embase and Cochrane Library) was conducted from each database’s inception to January 2023. Google Scholar was also searched, and search results were sorted by relevance. Grey literature such as blog posts, unpublished papers, news articles, corporate website postings and conference proceedings were not included as they mainly focused on international studies. A Google search was conducted to identify grey literature. Still, the results returned were mostly corporate websites, conference materials (e.g., abstracts, presentations) with no full text available, online news articles, or recruitment advertisements.

The search strategy only contained terms relevant to ‘care coordination’ and ‘chronic disease’, allowing us to maximise search yields and screen through articles relevant to our research aims. Medical Subject Headings (MeSH) terms were used, and the exact terms used are summarised in Appendix B.

### 2.2. Study Inclusion and Exclusion Criteria

Broadly, quantitative and qualitative articles were included if the study was conducted in Singapore and were programmes that involved care coordination for patients with chronic conditions defined under the Chronic Disease Management Programme (CDMP) but not patients at the end-of-life or undergoing palliative care due to the distinctly different requirements in managing this group of patients. Briefly, the CDMP is a list of the highest-burden chronic conditions in Singapore, with 23 of them at the time of this review [24]. The list of CDMP conditions is mentioned in Appendix C. The complete exclusion criteria are listed in Appendix D. 

### 2.3. Study Selection, Data Extraction and Analysis 

The search strategy was applied to all five databases and the identified articles were exported and managed in Microsoft Excel. Two independent reviewers (JYY and ACSL) assessed the eligibility of articles for inclusion through a two-stage screening process based on the Cochrane scoping review guidelines. In the first stage, the title and abstract of the articles were reviewed. Full-text articles were reviewed in the second stage. The screening was done independently by both reviewers to minimise potential bias. At the end of each stage, both reviewers discussed agreement on their ratings. Additional points of contention were clarified on an ad hoc basis. Reviewers’ recommendations were indicated using a three-point scale “yes”, “no” and “maybe”. For articles rated as “maybe”, reviewers appraised the articles again. The consensus was achieved after discussion without needing a third reviewer as a tiebreaker. Agreement was measured using Cohen’s weighted Kappa. 

Data on authors, year of publication, sample characteristics and specific details on care coordination models were extracted and detailed in Table 1 below. Thematic analysis of data sets was used to identify the subthemes that emerged from the programmes, focusing on care coordination elements.

However, as the programmes are highly pluralistic, the team required a framework to anchor the initial thematic analysis to facilitate the analysis process. To that end, the PESTLE framework was employed to rapidly identify the political/organisational, economic, sociocultural, technology-related/technical, legal, and environmental aspects in Table 2. Broadly, we operationalize political/organizational dimensions as elements that capture political, leadership or structural factors that facilitate the running of care models or programmes, economic dimensions as fiscal resources or financial commitments that go into the operationalisation of a programme, sociocultural dimensions as behavioural factors between stakeholders and associated perceptions harboured by stakeholders, technological/ technical dimensions as technological utilities and human resource capacities that enhance the efficiency of a programme and environmental dimensions as ecological factors that are beyond the individual stakeholder that often involve changes at the meso and macro levels. No legal factors were identified from the articles reviewed, and hence will not be discussed. This framework was chosen as it is widely used to guide logical and strategic examination of a specific programmatic issue and can be used to generate a deeper understanding of various elements surrounding organisational changes [25]. 

Fundamentally, we performed a validated multi-step approach to execute the framework analysis which comprises of the steps: (1) familiarisation of data sets, i.e., existing literature extracted, (2) identifying a framework, i.e., PESTLE, (3) coding and indexing, (4) charting by arranging indexed data into a framework matrix and (5) mapping and interpretation to obtain a representation of each key subtheme [26]. The emerging subthemes obtained from the framework analysis were subsequently organised into cross-cutting key themes which reached thematic saturation [27]. The emerging themes served as the foundational dimensions drawn from care coordination activities in Singapore.

**Table 1:** Characteristics of articles reviewed.

Author and Year of Publication	Activities Conducted	Mean Age of Study Participants	Eligibility Criteria	Aim of Programme	Financing Model	Cost to Patient	Training of Care Coordinators	Team Composition	Care Setting	Type of Chronic Disease	Length of Follow-Up	Mode of Contact	Frequency of Follow-Up
Ang et al. [28] (2019)	Book appointments Provide programme information Provide financial counselling Referral to appropriate resources	58 ± 16 years	Patients with chronic conditions who had at least 1 NUH SOC appt in the past year Clinically stable to be right-sited	Reduce hospital resource utilisation (Right-siting of care)	Not reported	Not reported	Not reported	Specialist physician + Primary care physician + Nurse + Allied health professional	Both Primary care & Hospital	Mixed	Not reported	Face-to-face	Not reported
Chandran et al. [29] (2013)	Assess clinical needs Assess medication compliance Conduct case finding Deliver disease-specific education Document care in electronic medical record systems Monitor patient’s progress Provide programme information Refer to appropriate resources Reinforce treatment compliance Screen for eligibility to enrol	72 ± 10 years	Aged > 50 years old History of low trauma fragility fracture Agree for at least 2 years follow-up	Optimise disease management	Not reported	Not reported	Nurse	Specialist physician	Hospital (Outpatient + Inpatient)	Osteoporosis	2 years	Phone call + Face-to-Face	6 follow-ups over 2 years
Chow et al. [30] (2014)	Act as a resource point for patients to call in for assistance/clarification Deliver tailored disease-specific education Deliver psychoeducation Deliver self-management education Escalate to physician Identify red flags Monitor patient’s progress Provide psychosocial support	63 ± 13 years	Patients aged ≥21 years old Recently discharged for diabetes-related hospitalisation with HbA1c >7% Agree to phone follow-ups	Reduce hospital resource utilisation (Transition from ward to home)	Not reported	Not reported	Nurse	Specialist physician	Hospital (Outpatient)	Diabetes	6 months	Phone call	4 follow-ups over 6 months
George et al. [31] (2016)	Deliver self-management education Identify red flags Monitor patients’ progress Provide smoking cessation counselling Reinforce appointment and medication compliance	73 ± 10 years	Patients clinically diagnosed with COPD or possible COPD Agree to be enrolled in a Disease Management Programme	Optimise disease management	Not reported	Not reported	Not reported	Specialist physician + Primary care physician	Both Primary care & Hospital	Chronic Obstructive Pulmonary Disease	Not reported	Phone call	Weekly or every 2 months depending on acuity
Ha et al. [32] (2020)	Advocate for patients’ preferences Collaborate with other care providers and with caregivers Deliver patient education Develop personalised care plans Liaise with and coordinate care processes between various stakeholders Empower caregivers Referral to appropriate resources	Not reported	Disease severity Extent of caregiver support	Reduce hospital resource utilisation (Right-siting of care), support caregivers and optimise disease management	Funded by MOH	No charges to patient	Not reported	Specialist physician + nurse + allied health professional + primary care physician	Both Primary care & Hospital	Dementia	Not reported	Phone calls + home visits	Not reported
Ha et al. [33] (2020)	Acts as a resource point for patients to call in for assistance/clarification Deliver patient education Monitor patients’ progress	78 ± 7.72 years	Patients with dementia identified with behaviours deemed challenging by caregivers and/or caregivers with caregiver burden Condition not stable/not suitable to receive care in the ambulatory dementia clinic	Reduce hospital resource utilisation (Right-siting of care) and support caregivers	Partially charity dollar funded	Yes	Not reported	Specialist physician + case manager + community-based counsellors	Off-site (ambulatory community clinic + at home)	Dementia	4 months	Home visits	Not reported
Jafar et al. [34] (2016)	Identify red flags Provide psychosocial support Reinforce treatment compliance	66 ± 10 years	Patients ≥ 40 years old Diagnosed with hypertension Uncontrolled BP Visited a participating polyclinic ≥ 2 times in the past 1 year Have no admission 4 months prior to recruitment Agreed to be enrolled	Optimise disease management	Not reported	Not reported	Nurse	Specialist physician + Primary care physician + Nurse	Primary care	Hypertension	3 months	Phone call	2 follow-ups over 3 months
Jafar et al. [35] (2022)	Coordinate with physician for care Collect requisite outcome data (clinical + patient reported health status) Empower patients Monitor patients’ progress Reinforce treatment compliance	64.5 ± 9.8 years	Patient aged ≥40 years with previously diagnosed hypertension Visited an enrolled clinic ≥2 times during prior 12 months Singaporean citizens or permanent residents	Optimise disease management	Partially subsidised by MOH	Yes	Nurse	Primary care physician + nurse + research coordinator	Primary care	Hypertension	2 years	Phone call	Monthly for first 3 months, then 3-monthly thereafter
Jiang et al. [36] (2019)	Conduct home visits Coordinate appointments Deliver disease-specific education Monitor patients’ progress Promote self-care Use of mobile application to educate, monitor and engage with nurse if needed	69.7 ± 11.04 years	Patients aged ≥ 21 years old Clinically diagnosed with chronic heart failure Able to read and understand English or Chinese Owned and used smartphones in their everyday lives Able to be followed up at home after discharge from hospital	Optimise disease management through self-care	Not reported	Not reported	Nurse	Nurse	Off-site (post-discharge)	Chronic heart failure	6 weeks	Home visits	Bi-weekly
Lai et al. [37] (2019)	Assess clinical needs Assess caregiver needs Conduct home visits Identify red flags Refer to appropriate resources	Not reported	Patients aged ≥ 65 years old and their caregiver Had uncomplicated memory loss for ≥6 months	Optimise disease management	Not reported	Not reported	Not reported	Specialist physician + Primary care physician + Nurse + Allied health professional	Primary care	Dementia	Not reported	Phone call + Home visits	Not reported
Lee et al. [38] (2015)	Act as a resource point for patients to call in for assistance/clarification Assess clinical and social needs Assess compliance to care plans Assess level of health literacy Assess medication compliance Coach caregivers and assess their competency Conduct home visits Coordinate follow-up visits with specialist care providers Deliver patient education Identify red flags Monitor patients’ progress Provide medication reconciliation Provide psychosocial support Refer to appropriate resources	68 ± 15 years	Patients aged > 21 years old Recently discharged home with high readmission risk ≥2 unplanned admissions within the past 90 days	Reduce hospital resource utilisation (Transition from ward to home)	Not reported	Not reported	Nurse with specialised training in care coordination	Specialist physician + Primary care physician + Nurse + Allied health professional	Off-site (Post-discharge only)	Mixed	3 months	Phone call	Weekly
Lim et al. [39] (2015)	Act as a resource point for patients to call in for assistance/clarification Collect data for programme evaluation Coordinate appointments Counsel patients on care process Liaise with and coordinate care processes between various stakeholders Monitor communication gaps between stakeholders Provide financial counselling Track prescriptions	54 ± 14 years	Patients who attend a tertiary rheumatology clinic Clinically stable to be right-sited	Reduce hospital resource utilisation (Right-siting of care)	Not reported	Not reported	Not reported	Specialist physician + Primary care physician + Nurse	Hospital (Outpatient)	Musculoskeletal disease	Not reported	Phone call	Not reported
Lim et al. [40] (2018)	Act as a resource point for patients to call in for assistance/clarification Book appointments Coach caregivers Coordinate with physician for care Coordinate transfer of care from SOC to Family Medicine Clinic Monitor patients’ progress Provide psychosocial support Recruit eligible patients to programme	64 ± 14 years	Patients with stable chronic diseases on follow-up at Family Medicine Clinic Patients with complex care needs on shared care between hospital and Family Medicine Clinic	Reduce hospital resource utilisation (Right-siting of care)	Not reported	Not reported	Nurse	Not reported	Primary care	Mixed	Not reported	Phone call	Not reported
Low et al. [41] (2017)	Act as a resource point for patients to call in for assistance/clarification Assess caregiver competency Assess compliance to care plans Assess level of health literacy Assess medication compliance Coordinate follow-up visits with specialist care providers Deliver patient education Enable patient activation Identify red flags Monitor patients’ progress Provide tailored care planning Referral to appropriate resources	71 ± 14 years	Patients aged ≥ 21 years old Have high risk of readmission Have ≥ 1 admission in the past 90 days Planned for discharge home Admitted in participating wards	Reduce hospital resource utilisation (Transition from ward to home)	Not reported	Not reported	Nurse	Specialist physician + Primary care physician + Nurse + Allied health professional	Off-site (Pre- and post-discharge)	Mixed	3 months	Phone call + Home visits	Weekly
Low et al. [42] (2015)	Act as a resource point for patients to call in for assistance/clarification Coordinate care with hospital specialists Conduct home visits Conduct medication reconciliation Deliver self-management education Monitor patients’ progress Provide nursing care Provide recommendations for physician reviews Refer to appropriate resources	Not reported	Patients with sub-acute or ≥3 chronic conditions requiring follow-up, or those with limited mobility that restricts access to healthcare services Not enrolled in other transitional care interventions ADL independent Do not have a caregiver at home	Reduce hospital resource utilisation (Transition from ward to home)	Fee for service	Yes	Nurse	Specialist physician + Primary care physician + Nurse + Allied health professional	Off-site (Post-discharge only)	Mixed	6 months	Phone call + Home visits	Not reported
Mustapha et al. [43] (2016)	Assess clinical needs Coach caregivers Coordinate family conference Deliver disease-specific education Monitor patients’ progress Provide psychosocial support Refer to appropriate resources	Not reported	Patients with chronic diseases or have end-of-life care issues	Reduce hospital resource utilisation (Transition from ward to home)	Not reported	Not reported	Nurse with specialised training in care coordination	Not reported	Off-site (Pre- and post-discharge)	Mixed	Not reported	Phone call	Not reported
Nurjono et al. [44] (2019)	Act as a resource point for patients to call in for assistance/clarification Assess clinical and social needs Coach caregivers Develop personalised care plans Deliver psychoeducation Manage patients’ social issues Monitor patients’ progress Promote self-care Provide psychological support Refer to appropriate resources	Not reported	Patients who are elderly and/or with complex healthcare needs Diagnosed with multiple chronic conditions Have limited mobility Presence of a caregiver at home	Reduce hospital resource utilisation (Transition from ward to home)	Funded by MOH	Yes	Nurse	Not reported	Off-site (Post-discharge only)	Mixed	3–12 months	Phone call	Not reported
Prabhakaran et al. [45] (2019)	Assess clinical needs Deliver tailored psychoeducation Develop tailored care plan Empower patients Identify red flags Monitor patients’ progress Referral to appropriate resources Reinforce compliance to care plans	37 ± 13 years	Patients aged ≥ 21 years old Discharged from ED with poorly controlled asthma Did not have complex comorbidities Own a mobile phone and able to use SMS Agree to enrol in the programme	Optimise disease management	Not reported	Not reported	Nurse	Not reported	Hospital (Outpatient)	Asthma	3 months	Phone call + algorithm-based automated text messages	Ad hoc
Verma et al. [46] (2012)	- Coordinate resources and services - Deliver psychoeducation - Identify strengths and resources and improve coping skills - Mediate or negotiate with stakeholders on patients’ behalf - Provide crisis management - Provide supportive counselling - Refer to appropriate resources - Set care goals - Support stress management	27 ± 7 years	Patients aged 16–40 years old Have first-episode psychotic disorder, not secondary to substance abuse or medical conditions	Optimise disease management	Funded by MOH	Not reported	Combination of nurse and non-nurse	Specialist physician + Primary care physician + Nurse + Allied health professional	Hospital (Outpatient)	Psychosis	2 years	Phone call	Not reported
Wee et al. [47] (2014)	- Coach patients and families - Collaborate closely with hospital physicians to plan and deliver care - Conduct home visits - Develop care plan - Enable self-management - Monitor patients’ progress - Refer to appropriate resources - Screen eligibility for programme enrolment	79 ± 8 years	Elderly adults discharged home Have complex care needs Have limited social support Not on follow-up with other case management or disease management programmes	Reduce hospital resource utilisation (Transition from ward to home)	Funded by MOH	No	Combination of nurse and non-nurse	Specialist physician	Off-site (Pre- and post-discharge)	Mixed	2 months	Phone call + Home visits	Not reported
Wee et al. [48] (2015)	- Conduct home visits - Deliver psychoeducation - Develop tailored care and medication plan - Monitor patients’ progress - Refer to appropriate resources - Screen eligibility for programme enrolment	Not reported	Elderly adults discharged home Have complex care needs Have limited social support Not on follow-up with other case management or disease management programmes	Reduce hospital resource utilisation (Transition from ward to home)	Funded by MOH	No	Not reported	Not reported	Off-site (Pre- and post-discharge)	Mixed	2 months	Phone call + Home visits	Not reported
Wong et al. [49] (2019)	- Act as a resource point for patients to call in for assistance/clarification - Collaborate with other care providers and with family members - Conduct home visits - Deliver psychoeducation - Deliver self-management education - Facilitate hospital admission - Monitor patients’ progress - Provide crisis management and support - Provide psychosocial support - Refer to appropriate resources	Patients: 27 ± 5 years Caregivers: 51 ± 14 years	Patients aged 16–40 years old Diagnosed with first-episode psychotic disorder, not secondary to substance abuse or medical conditions	Optimise disease management	Not reported	Not reported	Not reported	Specialist physician + Primary care physician + Nurse + Allied health professional	Hospital (Outpatient)	Psychosis	3 years	Phone call + Home visits	Not reported
Wong et al. [50] (2019)	- Advocate for patients’ preferences - Conduct home visits - Deliver psychoeducation - Develop tailored care plan - Empower patients and encourage strength building - Partner with other care providers and/or caregivers - Provide psychosocial support - Mediate conflict between patient and caregiver - Monitor patients’ progress - Refer to appropriate resources - Support crisis management	Case managers: 37 ± 9 years	Patients aged 16–40 years old Diagnosed with first-episode psychotic disorder, not secondary to substance abuse or medical conditions	Optimise disease management	Not reported	Not reported	Combination of nurse and non-nurse	Specialist physician	Hospital (Outpatient)	Psychosis	3 years	Phone call + Home visits	Not reported
Wong et al. [51] (2016)	- Act as a resource point for patients to call in for assistance/clarification - Deliver psychoeducation - Monitor patients’ progress - Identify red flags - Provide psychosocial support - Refer to appropriate resources - Reinforce compliance to care plans and medication - Screen eligibility for right-siting of care	59 ± 10 years	Post-elective Percutaneous Coronary Intervention patients Did not have a myocardial infarction 2 months prior to enrolment	Reduce hospital resource utilisation (Right-siting of care)	Not reported	Not reported	Nurse	Specialist physician	Off-site (Pre- and post-discharge)	Coronary artery disease	Not reported	Phone call	Not reported
Wu et al. [52] (2015)	- Assess clinical needs - Collect data for programme evaluation - Deliver psychoeducation - Develop tailored care plan - Encourage influenza vaccination - Liaise between step-down care partners, primary care physicians and patients - Offer advance care planning - Optimise patients’ medication regime - Refer to appropriate resources - Reinforce compliance to care plan	Not reported	Patients ≥ 40 years old Current/ex-smokers Have persistent/recurrent COPD-related respiratory complaints Not enrolled in other disease management programmes	Optimise disease management	Not reported	Not reported	Not reported	Specialist physician + Primary care physician + Nurse + Allied health professional	Off-site (Post-discharge only)	Chronic Obstructive Pulmonary Disease	Not reported	Phone call	Not reported
Wu et al. [53] (2018)	- Assess clinical needs - Coordinate appointments - Empower patients - Encourage influenza vaccination - Deliver psychoeducation - Deliver self-management education - Offer advance care planning - Optimise patients’ medication regime - Refer to appropriate resources - Reinforce compliance to care plan	72 ± 9 years	Patients ≥ 40 years old Current/ex-smokers Have persistent/recurrent COPD-related respiratory complaints Not enrolled in other disease management programmes	Optimise disease management	Funded by MOH	Not reported	Not reported	Specialist physician + Primary care physician + Nurse + Allied health professional	Off-site (Post-discharge only)	Chronic Obstructive Pulmonary Disease	Not reported	Phone call	Every 3–4 months
Yeo et al. [54] (2012)	- Facilitate transition between care settings - Monitor patients’ progress - Participate in care quality audit - Provide programme information - Refer to appropriate resources	Not reported	Diabetes patients seen at SGH Diabetes Centre Clinically stable for right-siting of care	Optimise disease management	Not reported	Not reported	Not reported	Specialist physician	Hospital (Outpatient)	Diabetes	1 year	Phone call	Not reported
Xu et al. [55] (2022)	Health and geriatric assessment Health coaching for disease prevention Chronic disease monitoring Education on self-care, medication and disease management Advanced care planning facilitation Refer to community health and social care agencies	71.4 ± 10.6	Patients with multiple chronic conditions Patients aged ≥ 60 years old Require assistance managing their chronic conditions	Reduce hospital resource utilisation (Transition from ward to home)	Not reported	Not reported	Nurse	Nurses with different experiences and qualifications	Off-site (community nursing post or patient home)	Mixed	2 years	Phone calls + home visits	Not reported

**Table 2:** Table of subthemes that are mapped into the PESTLE framework.

(P)olitical/ organizational -Clarity of programme direction and agenda setting -Standardisation of care model and processes across levels of care and at the national level -Support and commitment from senior leadership -Provider stakeholder interests and willingness to run the programme -Presence of programmatic champions
(E)conomic -Financial gradients across care settings for patients -Availability of resources to support programme implementation and staff development
(S)ociocultural -Quality of partnership with other care providers within the team and across different care settings -Interdisciplinary collaboration to deliver team-based care across care settings -Patients’ motivation towards the uptake of care at primary or community levels -Patients’ perceptions and understanding of services on offer -Quality of partnership between providers and patients and/or caregivers
(T)echnology/technical -Use of information systems that link patient data across care entities -Availability of alternative forms of communication between providers across various entities -Existing professional and technical capacities of providers in the community -Training opportunities and upskilling of care coordinators
(E)nvironmental -Flexibility to develop localised practices or protocols that suit contextualised needs -Proximity of provider location that can facilitate collaboration

## 3. Results 

### 3.1. Summary of Article Characteristics

Through systematically reviewing available articles, this review distilled 28 articles relevant to care coordination in the Singapore context summarised in Table 1 [28,29,30,31,32,33,34,35,36,37,38,39,40,41,42,43,44,45,46,47,48,49,50,51,52,53,54,55]. Out of the articles included, care coordination was specifically examined as part of the research question in four articles [43,44,49,50], of which two articles presented the perspectives of care coordinators [44,50], whereas 24 articles comprised relevant information on care coordination for a spectrum of programmes as part of broader interventions. A complete summary of the characteristics of all 28 articles reviewed is appended in Table 1.

The articles reviewed were published between 2012 and 2022. No eligible articles were published between 2010 and 2012. This suggests a greater focus on studying care coordination and related initiatives in recent years due to the unveiling of the *Three Beyonds* national strategy and the need to contain rising healthcare costs brought about by increased tertiary care usage.

### 3.2. Operational Characteristics of Programmes Reviewed

The aims of the programmes vary. A handful of programmes described a focus on lowering hospital resource utilisation by supporting patients during care transitions (36%) [28,31,39,42,43,45,46,50,55,56], and facilitating referrals from the specialist outpatient clinics (SOCs) to primary care providers (PCPs) (18%) [28,39,40,51,54]. The remaining 13 articles (46%) aimed to optimise the management of specific chronic diseases [29,31,32,33,34,35,37,45,46,49,50,52,53]. Importantly, heightened emphasis on reducing hospital resource utilisation and facilitating care transitions to lower levels of care aligns with the local vision of shifting care *Beyond Hospital to Community*. 

All programmes require a sustainable form of funding. Only six articles (21%) reported a financing model [42,44,46,47,48,53] and all except one, by Low et al., explicitly indicated funding from MOH [42]. In only two out of six articles, by Low et al. and Nurjono et al., reported that patients were required to pay fees, mainly for providers to conduct home visitations [42,44].

Building technical capacity by training new care coordinators is paramount for programme success. Nurses served as care coordinators in 46% of articles reviewed [29,30,34,37,38,40,41,42,43,44,45,51,55], out of which only 11% reported specialised training in care coordination [37,38,44], while only three articles cited a mix of staff with nursing and non-nursing backgrounds being equipped to discharge their care coordination duties [46,47,50]. 

The settings in which care services are offered play a crucial role in determining where, how and by whom the services will be provided. Congruent to shifting care *Beyond Hospital to Community*, most of the initiatives reviewed provide care off-site, i.e., via phone calls or at patients’ homes. Half of the articles reviewed depicted off-site care delivery [30,32,36,38,41,42,43,44,47,48,51,52,53,55], nine in the hospital setting [29,31,39,40,45,46,49,50,54] and three in primary care settings [34,35,37]. Only Ang et al. and Ha et al., reported care delivery occurring in both hospital and primary care settings under the same respective programme [28,33].

In addition, Singapore places heightened focus on key chronic diseases identified as high burden based on CMDP, as elaborated above. During the time of review, there were 23 chronic conditions listed under CDMP [24]. Nine out of 23 chronic conditions on the CDMP list were covered in the articles reviewed. A handful of articles (36%) described care coordination for a mix of chronic diseases [28,38,40,41,42,43,44,47,48,55]. 

Moreover, duplication of care may be a concern as patients with multiple chronic diseases may be enrolled in more than one care coordination programme. This imposes an additional burden on patients and their caregivers, who already interact with multiple healthcare providers. Our review found three articles (11%) reporting that patients enrolled in another ongoing care coordination programme were not eligible for the recruitment [43,52,54].

Importantly, continuity of care needs to be upheld in care coordination programmes. Robust follow-up processes reduce fragmentation of care as patients transition from hospital to community. Reviewed articles point to varying lengths of follow-up care. Most initiatives (57%) discussed were duration-bound and operated on follow-up periods spanning two months to three years [29,30,34,35,36,38,41,42,44,45,46,47,48,50,54,55]. 

Despite most care coordination models operating on a predominant disease-focused perspective, many articles also reported providing care coordination services to link patients to preventive care services and psychosocial support [30,36,38,40,43,44,45,46,49,50,51,52,53,55]. This reflects a shift *Beyond Healthcare to Health* as patients’ needs are being considered holistically. A broad view of the services conducted in the programmes is summarised in Table 1.

Crucially, activities such as process optimisation and care innovation allow care coordination to be delivered cost-effectively and longitudinally while working within the constraints of limited programme funding [39,44,45,52,54]. Besides improving internal processes, collaborations with appropriate care partners across the care interfaces enhance accessibility to care for patients residing in the community requiring a range of services and can contain the cost of healthcare delivery [31,32,35,37,39]. This enables a shift *beyond quality to value*.

### 3.3. Broad Mapping of Key Subthemes from Articles Reviewed

The subthemes from the PESTLE framework were distilled into seven cross-cutting main themes relevant to the operational success of care coordination of reviewed programmes in Singapore. 

***Standards and guidelines*** are fundamental in setting the overarching objectives and quality of care coordination initiatives. Having aligned a standardised set of guidelines on the model of care provision directly affects how programmes are implemented across institutions and improves programme fidelity. Heterogeneity in care models is evident from the diverse range of care coordination activities cited in the articles reviewed, even when comparing models managing patients with similar conditions and having similar programme goals. Some care coordination programmes had general disease-specific care management standards outlined by national working groups [29,36,46,53], while others developed standardised care and assessment protocols limited to individual institutions [34,44,45]. In contrast, an article by Wee et al., explicated that permitting flexibility in defining an institution-specific focus and processes facilitated care coordination, implying that differences in care models and standards should be tailored to suit the needs and culture of specific institutions or programmes [47].

Singapore harbours a repository of clinical practice guidelines for chronic diseases, but the recommendations largely focus on the clinical aspect of care. A Singapore study reflected the need to improve patient-centred care that encapsulates elements beyond the disease, emphasizing the need to cocreate guidelines and standards with providers from different care entities [56]. An absence of care coordination standards and their accompanying accredited guidelines creates operational challenges in programme implementation, especially when there is little clarity on the programmes’ expected outcomes and prospective lifespan [44,57]. However, it was unclear in most articles reviewed whether such standards were consistently implemented on the ground.

***Strong partnerships with other care providers*** can aid in fostering positive working relationships within the care coordination team to manage patient care holistically and effectively. Minting vertical and horizontal relationships are vital for successful care coordination workflows. Therefore, the strength of partnership and trust between providers in the community and hospital specialists impact the uptake of care coordination initiatives and alignment of outcomes for both providers and patients [41,47]. Moreover, leveraging partnerships between private and community stakeholders, streamlining referral processes and adopting collaborative care models encompassing mutually agreed upon right-siting workstreams empowers the shift of care *Beyond Hospital to Community* [32,33,37,39,44,46,51,54,55]. 

***An interoperable information system*** which can come in the form of a synchronised digital patient dashboard is a key catalyst that facilitates the linkage of patients and their data from tertiary to primary and community care points [33]. A study by Wee et al., evaluating various transitional care initiatives pointed to the utility of appropriate and integrated information systems to aid transitional processes across different care venues and gather data for programme evaluation purposes [48]. Furthermore, another article by Nurjono et al. reported that having multiple independent information systems at different care delivery points made sourcing for patient records by providers from different healthcare organisations a manually tedious process [44].

***Strong leadership*** surfaced as a prominent meso-level attribute for care services offered by various entities to be delivered optimally and collaboratively across various healthcare entities. Essentially, quality and consistency of leadership both within the organisation and at the individual programme level affect the implementation of care coordination services. A change in leadership and unclear programme direction was found to impact staff morale, especially regarding concerns about the future of their employment [44]. Leadership also heavily influences the amount of political and fiscal support for care coordination programmes in terms of strategic direction and resources. Different stakeholders within the hospital may have varying priorities and perceptions of care coordination, making it challenging to garner traction without strong leadership support to consolidate consensus when introducing new initiatives [48]. Getting buy-ins from organisational leaders signals organisational commitment to care coordination activities and aids in resolving role conflicts with other departments and programmes as highlighted by Chandran et al. and Ha et al. [29,33]. One study by Wee et al. had shown that the organisation readily channelled additional resources to fund the continuation of the care coordination programme after MOH funding ceased, which was only possible as senior leaders within the organization provided continued support for the initiative [48].

***Presence of resource availability*** determines the lifespan of the programme. Most care coordination programmes reviewed run on MOH programme funding, such that a fixed quantum allocated to the programme limits the duration and type of care model implemented. As a result, the lack of certainty for programme longevity influences the hiring of human resources, retention of manpower capacity and affects programme expansion [30]. This was reported to hinder access to multi-disciplinary expertise and for early review clinics to deliver responsive and holistic care as noted by Prabhakaran et al. [45]. Although community care providers are available, the perceived lack of technical and operational resources meant that care coordinators hesitated to refer their patients for continued care [44]. This observation may have resulted from the disparity in resources available at the hospital and community levels and hence led to a gap in the capacity to offer quality care [44]. On the other hand, Lai et al. discussed that collaborative care models with hospital specialists that comprise training modules and technical resources were introduced to upskill community providers to manage complex cases [37].

Another resource availability issue stems from the patient’s point of view, where the financial gradients between SOCs and private general practitioners (GPs) make the right-siting of care challenging [28,40,44]. Although the Community Health Assistance Scheme (CHAS), a portable medical subsidy scheme by the government that can be used at private GPs which provides additional subsidies for selected CDMP medications, the amount of financial subsidy was reported to be inadequate for CDMP patients who are on multiple medications to continue receiving long-term care within the private primary care sector [58]. 

***Patient-specific related factors*** include certain sociocultural factors, such as patients’ perceptions of certain care providers and unique motivations to seek care influence their acceptance of care coordination interventions [30]. For example, Ang et al. and Lim et al. highlighted that patients were disinclined to be referred to GPs to be followed up in the community if they viewed the quality of care as inferior to that provided in SOCs [28,39]. Patients’ understanding of care coordinators’ roles and capabilities also affects the perceived value of care coordination services [40,44]. This may result from infrequent contact between patients and care coordinators in traditional “more siloed” medical settings in Singapore, causing a lack of understanding of what care coordination entails [44].

Another patient-relevant factor is the presence of a supportive caregiver, especially for patients who are less independent in their daily activities. Care coordinators partner with this support network to monitor patients in the community by ensuring judicious follow-up attendance and medication reconciliation, among other responsibilities [32,43,49,50]. Mustapha et al. further reflected that simple care delivery and monitoring could be performed by trained caregivers who are competent care partners [43]. Williams et al. added that this reduces co-dependency on care coordinators and promotes care continuity after discharge from duration-limited care coordination programmes [59].

As many programmes harness patient empowerment through improved health literacy and self-efficacy, it is central to view care processes from the patients’ lens by considering their various commitments, the amount of effort required for behaviour change, and the physical barriers required to perform care tasks [36,60]. Most participants in care coordination programmes are reportedly older adults. They are more predisposed to suffer from multiple chronic diseases; hence, they may receive conflicting care instructions or be overwhelmed by different care regimens prescribed by multiple care providers [17].

***Provider-specific related factors*** include factors articulated from the providers’ point of departure. The presence of a passionate clinical stalwart was cited as a strong driver for programme success by bringing staff from within the organisation and across entities on board and overseeing their concerns [29,33]. Additionally, the proximity of location between providers and by having care coordinators embedded in the same practice venue were mentioned by Williams et al., as a facilitator that bolsters collaboration within the remit of care programmes [59]. As most care coordinators are sited in hospitals, this physical closeness allows hospital specialists to better understand the capabilities and duties of care coordinators and their roles in the patients’ journey. This colocation was highlighted to have enhanced the quality of their partnership by leveraging care coordinators to optimise the use of trans-disciplinary services, outside the ambit of a single speciality department, to meet the multi-dimensional needs of patients in the community [41,47,49].

Inarguably, care coordinators must be skilled in providing quality care coordination services and be comfortable communicating and operating across different levels of care and other care disciplines as noted by Vargas et al. [61]. While most care coordinators are nurses by profession, they cited challenges in delivering holistic care due to a lack of interdisciplinary training, especially related to the social aspects of care that extend beyond traditional nursing curriculums [44]. Fortunately, care coordinators function in multi-disciplinary teams. This means that care coordinators can refer older patients with more complex care needs to an appropriate teammate, such as a specialty nurse or social worker, to address the issues comprehensively within a readily available pool of expertise [37,43,50,55]. 

## 4. Discussion 

To our knowledge, this scoping review is the first to explore the various dimensions that facilitate care coordination in Singapore. This scoping review comprises 28 articles that painted a mixed picture of care coordination programmes but simultaneously revealed various cross-cutting themes that facilitate the care coordination of patients with chronic conditions in the community. Operationally, programmes largely focus on medical care with some dimensions of psychosocial support embedded in them, of which having a standardised set of guidelines was highlighted to improve the quality of care provided across settings. In terms of the operational features of these care models, most care coordinators have a nursing background and work in multi-disciplinary teams both within and across care interfaces. Fostering strong relationships between providers and strong leadership becomes an essential ingredient in collaboration across multiple care organisations so that all parties can manage patients in their respective care venues based on common goals. Having an interoperable information system further facilitates the movement of patients and their data across care settings more seamlessly. Data flow across care points is integral since patient communication often occurs via phone calls as care is generally delivered off-site. Care coordination programmes reviewed are often duration-limited and typically support patients for a period of two months to three years. Despite Singapore being heralded as a health system that offers quality healthcare, patient and provider expectations and perspectives can still hinder both parties’ willingness to manage patients or be managed at lower levels of care. An adverse financial gradient regarding subsidies received between the private and public sectors has also stymied the care-sharing process between public specialists and private primary care providers [62]. 

For any programme to operate optimally, forging a ***set of standardised guidelines*** is needed so that parties executing the programme have aligned principles to adhere to and enhance coherence throughout patient journeys. This is particularly important when faced with patients transitioning across various care levels and sectors, where institutional or organisational structure, financing and workplace culture might influence the quality of patient management [60,63]. Deploying a standard set of indicators and metrics can also provide windows for programmatic evaluations and identify gaps for improvement [64,65]. All programmes share a similar ambition of caring for patients in the community, but some aspects pertaining to the discharge of care coordination activities remain pluralistic, even for programmes that manage the same medical conditions. While programmes for different conditions may vary due to associated pathophysiology and care management requirements, a set of standardised guidelines need to be outlined to ensure consistency in the delivery of care coordination activities at multiple sites. This can be facilitated by adopting streamlined two-way referral processes, developing a collaborative model of care, and having an aligned fee structure agreed upon by all stakeholders along the care journey, embedded into the care programmes [44,66]. Guidelines have to be co-developed not only with fellow providers but patients as well to minimise potential gaps in the referral and right-sitting processes [67]. 

Conflicts between providers are not uncommon, especially across care entities [68]. Power dynamics between providers might also be a source of conflicting instructions and general dissatisfaction if left unresolved [69]. Installing ***strong leadership*** to spearhead such programmes is integral for ensuring that care offered to patients is not disrupted due to team management or interpersonal reasons [70]. Effective leadership is inherently entwined with the ability to cultivate resilient partnerships as mutual buy-ins for key stakeholders will promote commitment to common care coordination agendas [29,33]. Care quality can be enhanced when ***strong partnerships are forged between providers*** through intentional fostering of connections initiated by a programme champion [71]. This will strengthen inter-entity linkages and realise a collective agenda across care teams [72]. Multiple studies have shown that having a good pre-existing relationship is a facilitator for clear and collaborative communication channels for the delivery of care within a care team and across care entities [72,73]. 

Importantly, communication between providers can be heightened with ***an interoperable IT system***. In Singapore, the National Electronic Health Records (NEHR) platform enables patient data to be viewed and inputted across care entities. However, uploading patient data to the NEHR by private providers is not mandatory [74]. This lack of integration of patient data potentially hinders the effectiveness of transitional care programmes and stifles the right-siting of patients from public tertiary hospitals to private sector primary care providers, which comprise the majority of the primary healthcare landscape in Singapore [75]. An interoperable system that allows providers across different care levels to share patient data and communicate with each other in a real-time and data-secured manner should be explored [76]. 

Undoubtedly, ***resource availability*** such as medically relevant infrastructure, equipment and technically trained human resources are frequently viewed as key determinants to providing required care in any programme. However, community or primary care settings have often been seen as less well-resourced than higher care levels. The Singapore government has attempted to bridge this gap through various means, such as the family medicine clinics, which offer needed ancillary services to selected patients, such as diabetes and hypertension-related services [77]. More recently, the promulgation of primary care networks (PCN) has similarly enabled private GPs to do so. Such policy manoeuvres empower PCN care teams to dedicate resources such as nurse counselling, diabetic foot screening and diabetic retinal photography for diabetic patients in the community [62]. Therefore, having a stipulated financial and operational timeline will guide human resource hiring and assure that team-based care will be offered longitudinally to patients when sited in the community. Importantly, a few modifications to the delivery model can augment existing resources, such as replacing home visits with telehealth follow-ups where appropriate [29,30,31,34]. Effectively, telehealthcare can manifest as bringing care closer to patients’ homes, which aligns with the value that patients and caregivers place on convenience and access to care [78]. Such digitally enhanced services can also lower the overall cost to patients and maximise scarce medical resources [79]. 

Another way to minimise unnecessary healthcare expenditure and firmly anchor patients in the community is to educate and empower them to self-care. Emphasis on nurturing this ***patient-specific related factor*** can sow benefits not only for patients but also in terms of lowering provider burden and overall cost to the health system [80]. Research has shown that patients and their caregivers often feel ill-prepared to play a more substantial role in care management after discharge, due to perceived difficulties in managing the condition without direct professional involvement [81,82]. Effective approaches to patient engagement include adopting a patient-centred approach that supports self-management, which is linked to improved overall health outcomes and lower healthcare utilisation rates [83]. Additionally, the ability to self-care allows care to continue regardless of whether patients remain enrolled in a care coordination programme, thereby reducing dependency on care resources. Importantly, patient education on the need for care coordinators and the crucial roles they play in care transitions, need to be emphasised to inspire patient enrolment and adherence to regiments and instructions. Without a sociocultural mindset shift in expectations for these programmes, the desired outcomes might be hampered by a lower willingness to participate. 

Looking upstream, one of the ***provider-specific related factors*** includes providers’ professional training, which will impact their capacity to proficiently discharge care coordination duties. Therefore, care coordination should be taught more prominently in medical and nursing schools. Besides training clinical staff, expanding the reach to train non-clinical personnel to be care coordinators will grow the pool of skilled persons to perform care coordination activities. Such task-shifting can increase overall human resource capacity when specialised modules on care coordination for both clinical and non-clinical providers are implemented within and beyond the remit of care programmes [84]. Although not explicitly demonstrated in the articles reviewed, many studies point to nurses taking on the primary role of care coordinator on top of their regular duties [85,86,87]. Increased workloads might lead to burnout or a high turnover. Therefore, it might be timely for strategic task-shifting of care coordination duties to non-clinically trained personnel to feature more prominently [88]. 

Moreover, programmes that aim to have care coordination done well must not be fixated only on the medical elements but also the psychosocial [89]. Thus, it is pivotal to take a team-based care approach so that providers can refer patients to appropriately skilled providers to support patients beyond the traditional medical ambits during their care journey [90]. Simultaneously, moving away from a medically focused lens to a more holistic approach that embraces multi-disciplinarity (e.g., allied health and non-clinical personnel) which better addresses patients’ multi-faceted needs can increase the likelihood of patients remaining within the community [91]. However, there remains a dearth of literature on how care coordination across healthcare and social services differs from coordination within the realm of healthcare alone [63]. Although none of the reviewed articles illustrated how financial incentives can specifically drive the quality of care coordination process in Singapore, many other studies beyond Singapore had illustrated that a pay-for-performance financial incentive structure paid to providers for a particular service dimension, i.e., care coordination, warrants further exploration [92,93,94]. 

## 5. Limitations 

A limitation is that some programmes may not be covered as articles without full text and grey literature were not reviewed. A broader range of chronic diseases, beyond those on the CDMP list, will also enhance comprehensiveness of the review. 

## 6. Aligning with Singapore’s National Healthcare Strategies and Recommendations

As the national vision of the *Three Beyonds* paves the way for *Healthier SG*, more research needs to be done to investigate how other levels of care and sectors can be integrated into the primary healthcare system. There is an impetus to uncover the key facilitators for care teams to perform duties beyond the medical, to include other psychosocial aspects, such as social prescribing to fit into the mould of Singapore’s latest healthcare transformation, *Healthier SG*. To that end, care coordination processes need to be strengthened such that linkages between primary care providers and community partners are optimised. 

This scoping review has excavated the key themes pertinent to care coordination activities in Singapore. Firstly, this review has found several studies that describe how programmes are operationalised on the ground but left out the mechanisms that fund such programmes, including the exploration of optimal provider payment methods. Importantly, adverse financial gradients between public tertiary and private primary care providers need more exploration, as the CHAS subsidy quantum might not be sufficient for patients facing multiple chronic conditions to remain with their private primary care providers when they require more medications. The review also highlighted the need to understand the best methods to train providers in care coordination to manage an ever-growing and increasingly diverse patient population. Currently, most nurses play the role of care coordinator, and more needs to be done to augment the already clinically strained workforce. Crucially, while limiting care coordination services to a specified period may be a way to contain costs, little is known about the optimal duration for care coordination programmes. The frequency and duration of contact between patients and care coordinators may not be sufficient to build a meaningful therapeutic relationship and hence influence health outcomes beyond the programme duration [95]. Most importantly, the Singapore healthcare system requires more investigation of the numerous facets of care coordination and this review serves as a timely starting point.

## 7. Conclusions

This review highlighted the need for standards and guidelines, strong partnerships across stakeholders, interoperable information systems across entities, strong leadership, presence of resource availability, patient-specific factors such as sociocultural factors and provider-specific factors such as training, which can have an impact on care coordination when it comes to managing patients with chronic conditions in the community in Singapore.

Overall, care coordination activities are aligned with the national strategies, but implementation on the ground requires further refinement. With an ageing population and increasing chronic disease burden, more needs to be done to ensure effective and quality care coordination such that stable patients can be safely provided with the required care not only at the tertiary care level but also in the community. Lack of care coordination might cause patients to drift up the health system towards the hospital level. 

## Data Availability

These data sets will be made available by the research team only upon reasonable request.

## Appendix A. PRISMA-ScR Checklist

**Section**	**Item**	**PRISMA-ScR Checklist Item**	**Reported on Page #**
Title			
Title	1	Identify the report as a scoping review.	This has been stated in the introduction and methodology sections
Abstract			
Structured summary	2	Provide a structured summary that includes (as applicable): background, objectives, eligibility criteria, sources of evidence, charting methods, results and conclusions that relate to the review questions and objectives.	All these points are reported in the abstract.
Introduction			
Rationale	3	Describe the rationale for the review in the context of what is already known. Explain why the review questions/objectives lend themselves to a scoping review approach.	This is stated in the introduction section whereby the reason for this review is to surface key elements of care coordination as more patients with chronic diseases need to be firmly anchored in the community.
Objectives	4	Provide an explicit statement of the questions and objectives being addressed with reference to their key elements (e.g., population or participants, concepts and context) or other relevant key elements used to conceptualize the review questions and/or objectives.	This is mentioned in the introduction which aims to uncover the broad themes surrounding chronic disease management programmes with significant care coordination components while illuminating the under-researched areas requiring further exploration.
Methods			
Protocol and registration	5	Indicate whether a review protocol exists; state if and where it can be accessed (e.g., a Web address); and if available, provide registration information, including the registration number.	The entire review protocol is elaborated in the methodology section.
Eligibility criteria	6	Specify characteristics of the sources of evidence used as eligibility criteria (e.g., years considered, language and publication status), and provide a rationale.	This is fully mentioned in the methodology section.
Information sources	7	Describe all information sources in the search (e.g., databases with dates of coverage and contact with authors to identify additional sources), as well as the date the most recent search was executed.	This is fully mentioned in the methodology section.
Search	8	Present the full electronic search strategy for at least 1 database, including any limits used, such that it could be repeated.	This is fully mentioned in the methodology section and Appendix D.
Selection of sources of evidence	9	State the process for selecting sources of evidence (i.e., screening and eligibility) included in the scoping review.	This is mentioned in Appendix B and Appendix D.
Data charting process	10	Describe the methods of charting data from the included sources of evidence (e.g., calibrated forms or forms that have been tested by the team before their use, and whether data charting was done independently or in duplicate) and any processes for obtaining and confirming data from investigators.	This is fully mentioned in the methodology section and the PRISMA diagram shows how the team managed repeated and non-relevant articles.
Data items	11	List and define all variables for which data were sought and any assumptions and simplifications made.	This is highlighted in both the introduction and methodology sections which also includes our operational definition of care coordination.
Critical appraisal of individual sources of evidence	12	If done, provide a rationale for conducting a critical appraisal of included sources of evidence; describe the methods used and how this information was used in any data synthesis (if appropriate).	NA
Synthesis of results	13	Describe the methods of handling and summarising the data that were charted.	This is mentioned in the methodology section in terms of how data is extracted and thematically analysed by the study team. A PESTLE framework was used to guide the initial analysis.
Results			
Selection of sources of evidence	14	Give numbers of sources of evidence screened, assessed for eligibility, and included in the review, with reasons for exclusions at each stage, ideally using a flow diagram.	The inclusion and exclusion criteria are reported in Appendix B and in Figure 1.
Characteristics of sources of evidence	15	For each source of evidence, present characteristics for which data were charted and provide the citations.	The summary of programmatic characteristics is stated in the results section with the associated references.
Critical appraisal within sources of evidence	16	If done, present data on critical appraisal of included sources of evidence (see item 12).	NA
Results of individual sources of evidence	17	For each included source of evidence, present the relevant data that were charted that relate to the review questions and objectives.	The summary of programmatic characteristics is stated in the results section with the associated references and in Table 1.
Synthesis of results	18	Summarize and/or present the charting results as they relate to the review questions and objectives.	The summary of programmatic characteristics is stated in the results section with the associated references and Table 1.
Discussion			
Summary of evidence	19	Summarize the main results (including an overview of concepts, themes and types of evidence available), link to the review questions and objectives, and consider the relevance to key groups.	The summary of programmatic characteristics is stated in the results section with the associated references and Table 1.
Limitations	20	Discuss the limitations of the scoping review process.	This is discussed in the limitation section of the manuscript.
Conclusions	21	Provide a general interpretation of the results with respect to the review questions and objectives, as well as potential implications and/or next steps.	This is elaborated in the discussion and “Aligning with Singapore’s national healthcare strategies and recommendations” section.
Funding			
Funding	22	Describe sources of funding for the included sources of evidence, as well as sources of funding for the scoping review. Describe the role of the funders of the scoping review.	This is mentioned under the funding section.

## Appendix B. Sample Search Strategy Used for PubMed

### Appendix B.1. Steps Taken to Search Database

Step 1: Concepts 1–3 were searched individually.

Step 2: Results from Step 1 were combined by including a BOOLEAN term “AND” between search keywords for each concept.

### Appendix B.2. Search Filters Applied

1. Results by year: 2010–2023

2. Species: Humans

3. Language: English

**Number**	**Concept**	**Subject Headings**	**Search Keywords**
1	Care coordination	“Case Management” [Mesh]	care coordination OR care coordinator OR care coordinations OR care coordinators OR case management OR case manager OR case managers OR chronic care management OR chronic care manager OR care manager OR care managers
		“Transitional Care” [Mesh]	integrated care OR Integrated Health Care OR Integrated Delivery System OR Transitional Care OR care integration OR post-discharge care
		“Continuity of Patient Care” [Mesh]	Continuity of care OR patient handoff OR patient handover OR Patient transfer
		“Patient Care Management” [Mesh]	Patient Care Planning OR care management
		“Patient Navigation” [Mesh]	Patient Navigators OR Patient Navigator OR Patient Navigation OR Patient Navigations OR Care Navigator OR Care Navigators OR Health care navigator OR Health care navigators OR patient navigator program OR patient navigator programme
		“Delivery of Health Care, Integrated” [Mesh]	Integrated Delivery System OR integrated care OR care integration OR integrated health care OR health care integration
2	Chronic disease	“Chronic Disease” [Mesh]	Chronic diseases OR chronic disease OR chronic illness OR chronic illnesses OR chronically ill OR progressive disease OR progressive diseases OR progressive illness OR progressive illnesses OR chronic condition OR chronic conditions OR chronic disease management programme OR chronic disease management program OR CDMP
		“Comorbidity” [Mesh]	Comorbidity OR comorbidities OR comorbids OR comorbid OR multiple chronic condition OR multiple chronic conditions
		“Noncommunicable Diseases” [Mesh]	Noncommunicable disease OR Noncommunicable diseases OR non-infectious disease OR non-infectious diseases
2	Chronic disease	“Diabetes Mellitus” [Mesh]	Diabetic OR diabetics OR diabetes mellitus OR diabetes OR elevated blood glucose OR high blood glucose OR glucose intolerance OR pre-diabetes OR pre-diabetic OR pre-diabetics OR impaired fasting glucose OR impaired glucose tolerance
		“Hypertension” [Mesh]	hypertension OR hypertensive OR high blood pressure OR essential hypertension OR malignant hypertension OR elevated blood pressure OR elevated systolic
		“Lipid Metabolism Disorders” [Mesh]	Lipid Metabolism Disorder OR Lipid Metabolism Disorders OR dyslipidemia OR dyslipidemias OR high cholesterol OR high cholestrol OR lipid disorder OR lipid disorders OR high triglycerides OR hypercholesterolemia
		“Asthma” [Mesh]	Asthma OR Asthmas
		“Pulmonary Disease, Chronic Obstructive” [Mesh]	chronic respiratory disease OR chronic respiratory diseases OR COPD OR chronic obstructive pulmonary disease OR chronic obstructive pulmonary diseases OR bronchitis
		“Renal Insufficiency, Chronic” [Mesh] OR “Nephrosis” [Mesh]	Nephrosis OR nephrotic OR nephrotic syndrome OR chronic kidney disease OR kidney disease chronic OR kidney diseases OR kidney diseases OR chronic renal disease OR renal disease OR chronic renal diseases OR renal diseases OR nephritis OR nephropathy OR pyelonephritis OR chronic kidney insufficiency OR chronic renal insufficiency OR chronic kidney insufficiencies OR chronic renal insufficiencies OR nephrotic disease OR nephrotic diseases
		“Schizophrenia” [Mesh]	Schizophrenia OR Schizophrenias OR Schizophrenic Disorder OR Schizophrenic Disorders
		“Depressive Disorder” [Mesh]	Depression OR depressive disorder OR depressive disorders OR depressive syndrome OR depressive syndromes OR unipolar depression OR major depressive disorder OR major depressive disorders OR major depression
		“Bipolar Disorder” [Mesh]	Bipolar disorder OR bipolar disorders OR psychosis OR psychoses OR bipolar depression OR manic disorder OR manic disorders OR mania OR manias
		“Anxiety Disorder” [Mesh]	Anxiety disorder OR anxiety disorders OR anxiety
2	Chronic disease	“Stroke” [Mesh]	Stroke OR strokes OR brain ischemia OR brain ischaemia OR cerebrovascular accident OR cerebrovascular accidents OR cerebrovascular stroke OR cerebrovascular strokes OR cerebral stroke OR cerebral strokes OR acute stroke OR acute strokes OR cerebrovascular disease OR cerebrovascular diseases
		“Dementia” [Mesh]	Dementia OR dementias OR senile dementia OR Alzheimer disease OR Alzheimer’s disease OR Alzheimer diseases OR Alzheimer’s diseases OR vascular dementia
		“Osteoarthritis” [Mesh]	Osteoarthritis OR osteoarthrosis OR osteoarthroses OR degenerative arthritis OR degenerative arthritidies OR rheumatoid arthritis
		“Parkinson Disease” [Mesh]	Parkinson Disease OR Parkinson’s Disease OR Parkinsonism
		“Prostatic Hyperplasia” [Mesh]	Prostatic Hyperplasia OR benign prostatic hyperplasia OR benign prostatic hypertrophy OR prostatic hypertrophy
		“Epilepsy” [Mesh]	Epilepsy OR epilepsies OR seizure disorder OR seizure disorders OR epileptic syndrome OR epileptic syndromes OR epileptic disorder OR epileptic disorders OR chronic epilepsy
		“Osteoporosis” [Mesh]	Osteoporosis OR osteoporoses OR bone loss OR bone losses
		“Psoriasis” [Mesh]	Psoriasis OR psoriases OR psoriatic arthritis OR arthritic psoriasis OR psoriatic arthritis OR psoriatic arthropathy OR psoriatic arthropathies
		“Arthritis, Rheumatoid” [Mesh]	Rheumatoid Arthritis OR Sjogren’s syndrome OR rheumatoid vasculitis OR chronic inflammatory autoimmune disease OR chronic inflammatory autoimmune diseases
		“Cardiovascular Diseases” [Mesh]	Cardiovascular disease OR Cardiovascular diseases OR ischaemic heart disease OR ischemic heart disease OR myocardial ischemia OR myocardial ischaemia OR myocardial infarction OR ischaemic heart diseases OR ischemic heart diseases OR myocardial infarctions OR coronary arterial disease OR coronary artery disease OR coronary arterial diseases OR coronary artery diseases OR heart disease OR heart diseases OR coronary disease OR coronary diseases
3	Singapore	“Singapore” [Mesh]	Singapore OR Singaporean OR Singaporeans OR Chinese OR Malay OR Malays OR Indian OR Indians

## Appendix C. List of Chronic Disease Management Programme (CDMP) Conditions

1. Anxiety
2. Asthma
3. Benign Prostatic Hyperplasia
4. Bipolar Disorder
5. Chronic Kidney Disease (Nephritis/Nephrosis)
6. Chronic Obstructive Pulmonary Disease
7. Dementia
8. Diabetes mellitus and Pre-diabetes
9. Epilepsy
10. Hypertension
11. Ischaemic Heart Disease
12. Lipid Disorders
13. Major Depression
14. Osteoarthritis
15. Osteoporosis
16. Parkinson’s Disease
17. Psoriasis
18. Rheumatoid Arthritis
19. Schizophrenia
20. Stroke

## Appendix D. Exclusion Criteria Used to Screen Articles

**No.**	**Exclusion Criteria**	**Rationale**
1	Does not relate to and/or describe care coordination	Align with the review to consider initiatives that describe care coordination
2	Disease is not part of the CDMP list	Align with the review aim to examine initiatives related to management of chronic diseases on the CDMP list.
3	Initiative does not target population of interest (i.e., community-dwelling adults who are neither aged 21 and above nor have a chronic disease)	Care coordination for adults who are not community-dwelling or non-adult populations involves different considerations (e.g., liaising with the nursing home care team, partnering with parents and teachers).
4	Articles are not based on an initiative in Singapore	Align with the review aim to study initiatives in Singapore.
5	Articles not in English	Eliminate the risk of losing information due to translation issues.
6	Articles assessed and/or described an alternative form of intervention (e.g., acupuncture)	A sample of 50 articles on alternative therapies were reviewed and the articles did not contain any element and/or description of care coordination. Hence, this criterion was added midway through the stage 1 screening process.
Additional exclusion criteria for stage 2 screening		
7	Full text is not available	Abstracts may not provide a good representation of the initiative and sufficient level of detail, which does not allow for a comprehensive assessment of the article.
8	Care recipients belong to end-of-life population	The target population is the general Singapore population that is suffering from chronic diseases that require prolonged management in the community as compared to end-of-life populations that need a different set of care management requirements.
